# Evaluation of reducing the south and reinforcing the north method on postmenopausal atherosclerosis disease based on 4 diagnostic objectifications: A prospective observational study

**DOI:** 10.1097/MD.0000000000037615

**Published:** 2024-04-05

**Authors:** Hongjin Wu, Weiwei Dai, Shenguang Li, Liping Tu, Jiatuo Xu, Zhifeng Zhang

**Affiliations:** a Central Laboratory for Science and Technology, Longhua Hospital Shanghai University of Traditional Chinese Medicine, Shanghai 200032, China; b Shanghai Minhang Integrated Traditional Chinese and Western Medicine Hospital, Shanghai 201199, China; c Shanghai University of Traditional Chinese Medicine, Shanghai 201203, China.

**Keywords:** Four diagnostic objectification, Hyperlipidemia, Postmenopausal atherosclerosis, Reducing the south and reinforcing the north method

## Abstract

Reducing the south and reinforcing the north method (RSRN) has a positive effect on atherosclerosis disease. However, there is a lack of objective standards based on the quantification of 4 diagnostic methods in evaluating the improvement or effectiveness of the treatment. This study aimed to explore the quantitative evaluation of the therapeutic effect of RSRN on postmenopausal atherosclerosis based on the 4 diagnostic methods. The observational prospective cohort study was conducted at Longhua hospital Shanghai University of traditional Chinese medicine. According to the inclusion criteria, 96 patients (disease group) and 38 healthy cases (control group) were selected, the pulse parameters were compared between the 2 groups to demonstrate the reliability and success of the disease model. Then 4 diagnostic information before and after RSRN treatment were collected and statistical analyzed by 1-way analysis of variance (ANOVA) (with Bonferroni correction). Furthermore, social network analysis was used to analyze the changes of symptoms, tongue, pulse, and complexion characteristics before and after treatment. There was a significant difference in pulse parameters between the disease group and the control group. The pulse parameters *t*1, *h*3, *h*3/*h*1, *h*4/*h*1, *S*, As, and *w* values in disease group were higher than those in control group, while the *h*5, *h*5/*h*1, and Ad values were lower than those in control group (*P* < .05). After the treatment of RSRN, the clinical symptoms of patients were greatly improved. The facial color indexes *L, a, b* values of the disease group at week 6 were different from those at week 0 (*P* < .05). The overall brightness and chroma of the patient’s facial color were significantly improved. The patients had virtual string pulse at week 0, and mainly string I and string II at week 7. The pulse parameters *t*1, *t*5, *w, w*/*t, h*1, *h*5, *h*3/*h*1, and *h*5/*h*1 values at week 7 were different from those at weeks 0, 1, 2 (*P *< .05); the tongue image was mainly red and crimson, peeling or greasy fur at week 0, while at weeks 6, 7, mainly light red, or thin white tongue. The RSRN method can regulate the complexion, tongue and pulse condition, clinical symptoms of postmenopausal atherosclerosis.

## 1. Introduction

The postmenopausal women suffered from dyslipidemia due to the decline of ovarian function and the decrease of estrogen level, estrogen-related apolipoprotein synthesis and fatty acyltransferase activity decreased, which increased blood triglycerides and accelerated the formation of atherosclerosis (AS), lead to increasing incidence rate of AS and cardio cerebrovascular diseases in postmenopausal women.^[1]^ Arteriosclerosis is the core of the aging process of cardiovascular diseases, which reflects a variety of pathological features, including AS, vascular calcification and inflammation, end-stage renal disease, and vascular lesions.^[2]^ AS is the main pathological basis of cardiovascular and cerebrovascular diseases, which are characterized by high incidence rate, high disability rate, high mortality rate, and high recurrence rate. The 2021 annual meeting of the American College of Cardiology (ACC) pointed out that cardiovascular disease was the number one killer of women. There were about 275 million women suffered from cardiovascular disease, and about 9 million women worldwide died of cardiovascular disease according to the 2019 global burden of disease research data (GBD).^[3]^ It was reported that cardiovascular disease was still the main cause of death in postmenopausal women, and nearly 56% of deaths in Western European countries were caused by cardiovascular disease.^[4]^ Therefore, the prevention and treatment of AS is the main research direction and research hotspot in the prevention and treatment of menopausal diseases.

In recent years, the research of Chinese herbs has shown great advantages and potential in the prevention and treatment of postmenopausal diseases. The multi-component and multi-effect of Chinese herbs are consistent with the multi-factors and multi-links of AS. Therefore, it is necessary to find herbs to prevent and treat AS in postmenopausal women. However, due to the complexity of Chinese medicine, especially Chinese medicine compound, its specific mechanism is still not completely clear, so much research is needed.^[5]^ Reducing the South and reinforcing the North method (RSRN) is a treatment method proposed according to the characteristics of menopausal atherosclerotic diseases in traditional Chinese medicine (TCM).^[6]^ Previous clinical and basic experiments also showed that the main effective components of RSRN, with high content of flavonoids, has estrogen like effect, can regulate blood lipid metabolism, improve arteriosclerosis, and play the role of anti-postmenopausal AS.^[7–9]^ This study dynamically evaluated the efficacy of RSRN in the treatment of postmenopausal AS based on the objective indicators of the 4 TCM diagnostic methods, which fully reflected the characteristics and advantages of TCM in clinical practice.

**Figure 1.**
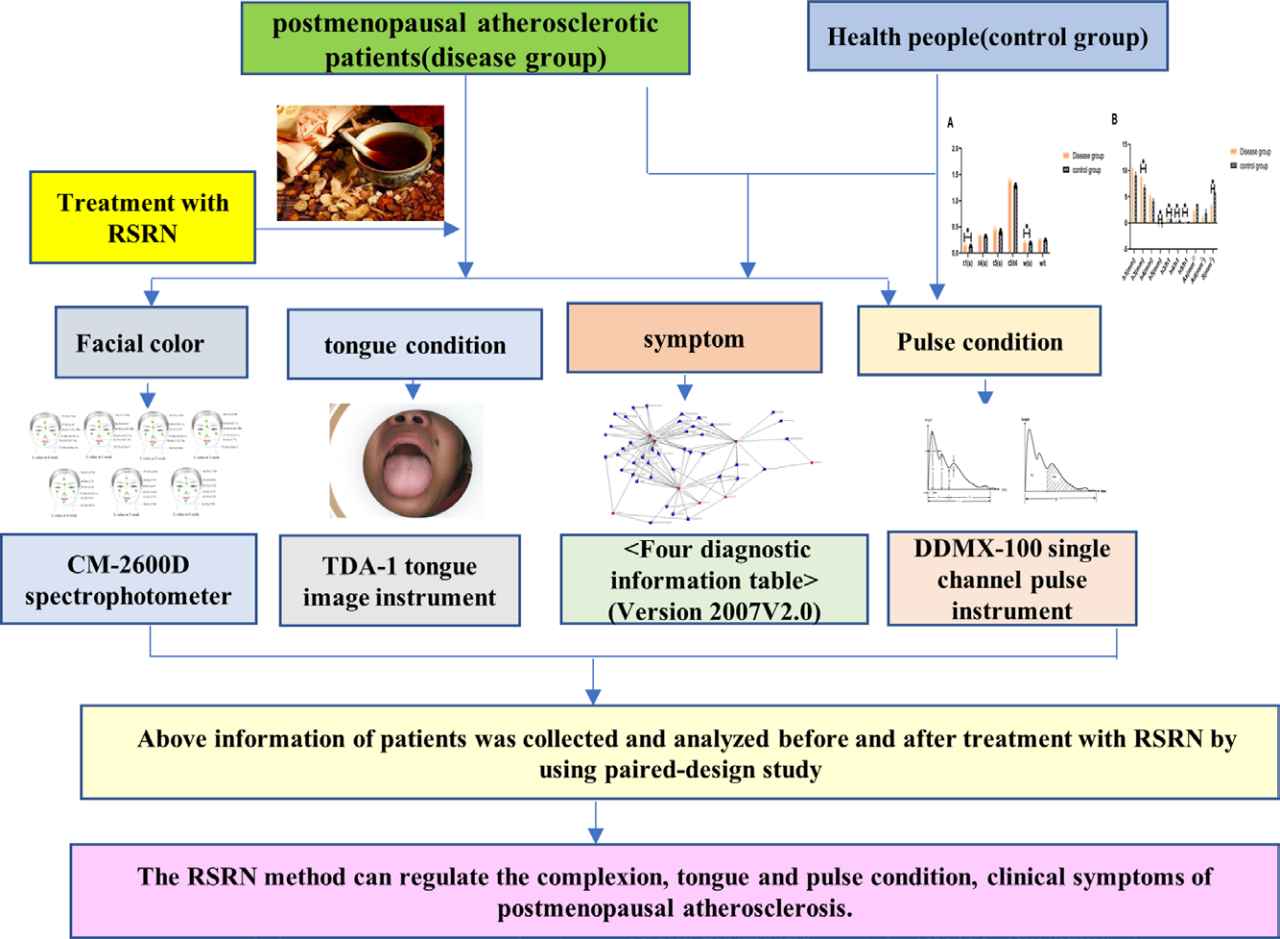
The implementation plan of the project.

TCM syndrome differentiation relying on subjective experience can be transformed into a combination of macro and microsyndrome differentiation by modern diagnostic techniques.^[10]^ At present, it is known that the complex characteristics of network biology coincide with the multi-channel, multi-target, integrated regulation mechanism and the elaboration of life law of TCM. The biological network regulation of syndromes and drug targets is obtained by constructing the biological network of TCM syndromes, which will provide new ideas for elucidating the essence of TCM syndromes and the intervention targets of compound Chinese medicine. Based on the theory of “combination of disease and syndrome,” the postmenopausal atherosclerotic patients were selected as the research objects, the changes of postmenopausal AS patients’ 4 diagnostic information characteristics during RSRN treatment were analyzed, and the treatment effect of RSRN on postmenopausal atherosclerotic disease was also explored. Furth more, the therapeutic effect of RSRN on postmenopausal atherosclerotic diseases was discussed. This research provides the possibility of using 4 diagnostic images and digital diagnostic information for efficacy evaluation (Fig. 1).

## 2. Materials and methods

### 2.1. Recruitment and characteristics of patients

The prospective cohort study was conducted. All patients were from Wujing Branch Longhua Hospital Shanghai University of TCM. Ninety-six postmenopausal atherosclerotic patients met the inclusion criteria were included into disease group (female, aged 47.8 [5.71] years); 38 healthy people from medical examination center were included into the control group (female, aged 30.24 [3.96] years). Information from the 4 diagnostic methods before and after treatment (from 0 to 7 week) in patients were collected and statistical analyzed.

This study was approved by the Medical Ethics Committee of Wujing Branch Longhua hospital Shanghai University of TCM. The patients gave informed consent and agreed to participate in the study. The study was in accordance with the Helsinki declaration and Chinese relevant clinical trial research standards and regulations. All subjects included in this study were introduced to the purpose and treatment methods by the researcher before collection of clinical information. This study was designed for observational research, and the Strengthening the Reporting of Observative Studies in Epidemiology checklist for research reporting of observational studies was followed in this study.

### 2.2. Diagnostic criteria

The early stage of the disease should be considered in elderly patients with dyslipidemia, vascular stenosis or dilated lesions found by X-ray, ultrasound, and arteriography.^[11]^ It can be diagnosed by X-ray, Doppler ultrasound, CT angiography, magnetic resonance angiography, and arteriography in the middle and late stage.

### 2.3. TCM syndrome differentiation criteria

1. Dizzy of the head and dim of sigh (tou yun, yan hua); dry eyes (muse); vision loss (shi li jian tui); costal burning pain (xie le zhuo tong)^[12–14]^;
2. Soreness or pain of waist and knee (yao xi suan ruan teng tong); loose teeth and hair loss (chi song fa tuo); tinnitus (er ming);
3. Hot flashes and night sweats (chao re dao han); zygomatic redness in the afternoon (wu hou quan hong); five heart upset heat (wu xin fan re); dry mouth and dry throat (kou zao yang an);
4. The tongue is red with little moss (she hong shao tai), and the pulse is thin (mai xi shu).

Liver and kidney yin deficiency syndrome can be diagnosed by (1) and (2) one or more symptoms +(3) one or more symptoms +(4).

### 2.4. Inclusion criteria and exclusion criteria

#### 2.4.1. Inclusion criteria.

Patients aged at 40 to 60 years old; met the diagnostic criteria; met the standard of syndrome differentiation; the patient was conscious, without aphasia and intellectual impairment, and could understand all the contents of the scale; those who signed the informed consent form and were willing to participate in the research.

#### 2.4.2. Exclusion criteria.

With acute infectious diseases; clearly diagnosed with respiratory, cardiovascular, cerebrovascular, liver and kidney, blood, endocrine and other system diseases; unwilling to accept research measures or unable to cooperate for other reasons.

### 2.5. Shedding cases and treatment

Standard of falling off: Qualified subjects who failed to complete the specified course of treatment and observation cycle for some reasons were regarded as shedding cases; treatment of abscission cases: When the subject fell off, the researcher shall contact the subject and complete the project as far as possible by means of door-to-door, telephone; and filled in the “case shedding reason table” and “treatment summary table” in the study case record form.

### 2.6. Herbal preparation and TCM diagnostic methods

#### 2.6.1. Herbal preparation.

**Figure 2.**
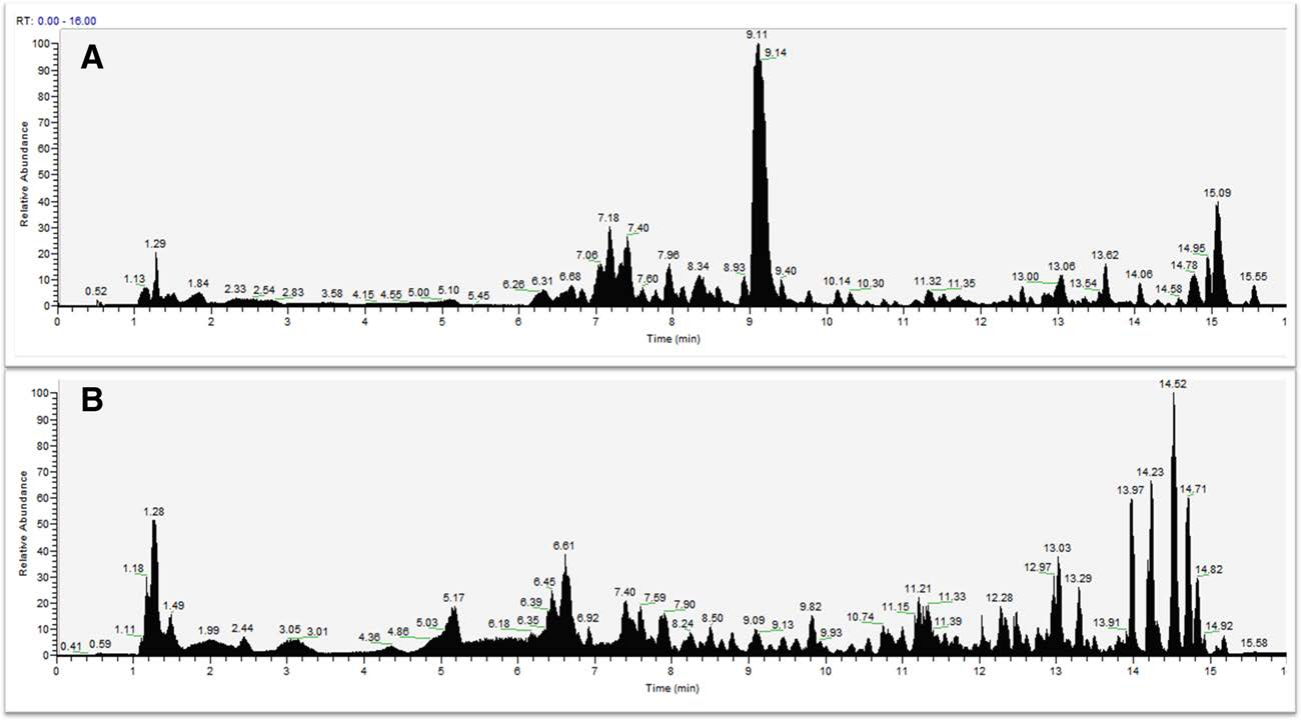
The ion flow chromatogram under the positive and negative ion modes of RSRN (A-positive mode; B-negative mode).

Curculigo orchioides Gaertn (Xian mao) 30 g, Epimedii Folium (Yin yang huo) 30 g, Morindae officinalis Radix (Ba ji tian) 15 g, Angelicae Sinensis Radix (Dang gui) 15 g, Phellodendron chinense Cortex (Huang bo) 12 g, Anemarrhenae Rhizoma (Zhi mu) 10 g, Salviae Miltiorrhizae Radix et Rhizoma (Dan shen) 12 g, Coptidis Rhizoma (Huang lian) 6 g. All herbs were obtained from Longhua Hospital Shanghai University of TCM, China. HPLC was used to determine the components in RSRN.^[9]^ The ion flow chromatogram under the positive and negative ion modes of RSRN was shown in Figure 2. The dosage and herbs should be added or reduced according to the syndrome in clinical application.

#### 2.6.2. Consultation index and method.

The <Four diagnostic information table > (Version 2007V2.0)^[15]^ was recorded before and after treatment. The records included the patient’s age, occupation, marital status, current medical history, pregnancy history, marriage history, medication history, clinical symptoms, tongue condition, pulse condition.

#### 2.6.3. Facial color examining device and methods.

The CM-2600D spectrophotometer (D50 light source, 2° visual angle) device was used to collect facial spectrum and color of different points (forehead, eyebrow, nose, chin, left zygoma, right zygoma). The selection and analysis of characteristic values for the facial color: CIE L*a*b* color space was used in this study.^[16]^ The L*value represents the luminosity, which means the lightness of color, ranging from 0 to 100 (absolute black to absolute white). The a* value runs in range of +127 to −128 (red to green) and the b* value suggests the range from yellow to blue with the value between +127 and −128 (yellow to blue).

#### 2.6.4. Tongue imaging device and methods.

Tongue diagnostic analysis-1 tongue image instrument (developed by the Intelligent Processing Laboratory of TCM diagnosis information of Shanghai University of traditional Chinese medicine) was used to collect tongue images. The software of “TCM Tongue Diagnostic Analysis System (TDAs) V2.0” was used for tongue image analysis.

The color of tongue images in TIFF format after color correction would be recognized by 3 TCM diagnosis professionals. Those that received the agreement from all 3 professionals would be included in this study. According to the literature’s reporting,^[17,18]^ tongue color (she zhi) would be classified into 4 types, namely, pale tongue (dan bai), light red tongue (dan hong), red tongue (hong jiang), and purplish tongue (qing zi) (including light purple tongue and blue and purple tongue); the color of tongue coating (she tai) would be classified into 4 types, white moss (bai tai), greasy moss (ni tai), yellow moss (huang tai), and peeled moss (bo tai). *L* (lightness), *a* (red green), *b* (yellow blue) value was selected as the characteristic tongue color parameters.^[16]^

#### 2.6.5. Pulse wave examining device and methods.

DDMX-100 single channel pulse instrument (Patent No.: ZL.200520038993.8) was used for collecting pulse waves on the area guan in left hand of patients by students who have been professional trained. Pulse classification: xian I (it is often seen in functional lesions, or early hypertension); xian II (it can be seen in hypertension and arteriosclerosis, indicating that the dilation function of artery and left ventricle is decreased); xian III (it is often seen in arteriosclerosis with increased peripheral resistance); xian IV (it is often seen in arteriosclerosis and decreased cardiac function); xu xian (it is often seen in qi and blood deficiency of heart); xian hua (It is often seen in autonomic nerve instability or disease development stage). The pulse classification was carried out according to the waveform and parameters of pulse graph, the index and classification standard of the pulse graph refer to the <modern traditional Chinese medicine pulse diagnostics > edited by Fei Zhaofu.^[19]^

**Figure 3.**
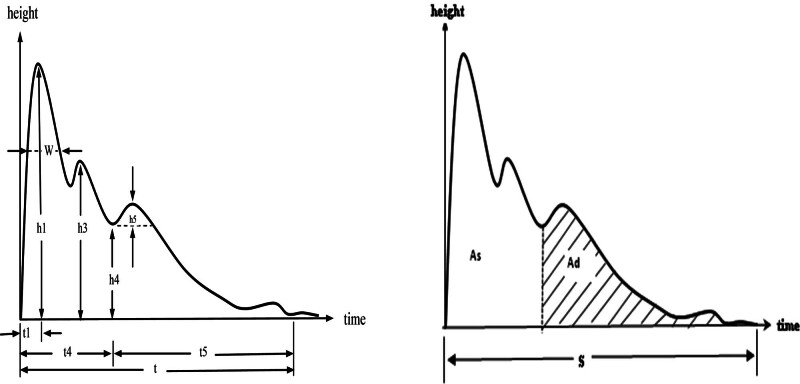
Pulse wave parameters.

The parameters of pulse wave (Fig. 3), *h*1 (main wave amplitude, reflecting left ventricular ejection function and arterial compliance), *h*3 (heavy wave amplitude, reflecting arterial elasticity and peripheral resistance), *h*4 (descending isthmus amplitude, reflecting arterial peripheral resistance), *h*5 (heavy wave amplitude, reflecting arterial elasticity and aortic valve function), *h*3/*h*1 (reflecting vascular wall compliance and peripheral resistance), *h*4/*h*1 (reflecting arterial vascular peripheral resistance), *h*5/*h*1 (reflecting aortic compliance and aortic valve function), *w* (1/3 width on the main wave, reflecting arterial elasticity and aortic valve function), *w*/*t* (reflecting arterial wall elasticity and peripheral resistance), *t*1 (time value of left ventricular rapid ejection phase), *t*4 (systolic time value of left ventricle), *t*5 (diastolic time value of left ventricle), *S* (total pulse area), As (systolic area), Ad (diastolic area).

### 2.7. Estimation of sample size

To determine the test set sample size, we calculated the sample size using the PASS 15 (Power Analysis and Sample Size Software, 2017; NCSS, LLC, Kaysville, UT, ncss.com/software/pass). Based on preliminary clinical observations.^[7,20]^ Setting *α* = 0.05 (bilateral), *β* = 0.10. The probability of making Class I errors in inferential conclusions (both sides) should not exceed 0.05, and the probability of making Class II errors should not exceed 0.10. It was inferred that the sample size of the disease group needs to be at least 45 cases.

### 2.8. Statistical methods

SPSS20.0 statistical software was used for statistical analysis. The normality test on each group of data was performed. Kolmogorov Smirnov test was employed to verify data distribution. The pulse parameters between disease and control group were analyzed by *t* test. The complexion index, pulse parameters, and tongue color parameters before and after RSRN treatment was statistically analyzed using 1-way analysis of variance (ANOVA) (with Bonferroni correction). The data were expressed as mean (SD) (mean ± standard deviation). The significant difference was expressed as *P* < .05.

### 2.9. Construction of 2-module network

We constructed the 2-model network model based on symptoms, pulse, and tongue condition at different weeks using the social network analysis software UCINET (University of California at Irvine network), and then analyzed the distribution characteristics and relationship of symptoms, pulse, and tongue condition.^[21]^ The multi-dimensional scale layout was used to draw the visual map of symptom/pulse/tongue condition-2-module network at different periods, to visually show the internal relationship between symptom/pulse/tongue condition at different periods.

## 3. Results

### 3.1. Results of pulse parameters between disease and control group

**Figure 4.**
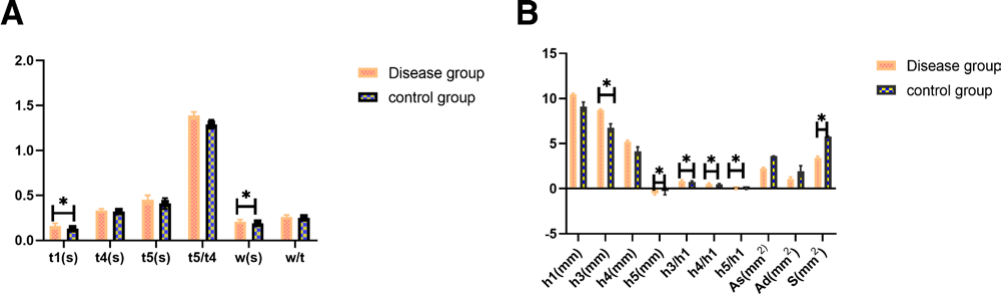
Results of pulse parameters between two groups. (A) Changes of pulse parameters *t*1, *t*4, *t*5, *t*5/*t*4, *w, w/t* between the disease group and the control group. The pulse parameters *t*1, *w* in the disease group were higher than those in the control group; (B) Changes of pulse parameters *h*1, *h*3, *h*4, *h*5, *h*3/*h*1, *h*4/*h*1, *h*5/*h*1, *As, Ad, S*. The pulse parameters *h*3, *h3*/*h*1, *h*4/*h*1 in the disease group were higher than those in the control group, *h*5, *h*5/*h*1 in disease groups were lower than those in the control group.

The pulse parameters *t*1 (0.16 [0.03]s), *h*3 (8.71 [4.11]mm), *h*3/*h*1 (0.83 [0.75]), *h*4/*h*1 (0.51 [0.11]), and *W* (0.21 [0.02]s) in disease group were significantly higher than those (*t*1 [0.13 {0.02}s], *h*3 [6.74 {2.00}mm], *h*3/*h*1 [0.75 {0.11}], *h*4/*h*1 [0.46 {0.09}], *w* [0.19 {0.03}s]) in the control group; while the pulse parameters *h*5 (−0.53 [0.55]mm), *h*5/*h*1 (−0.06 [0.05]) in the disease group were significantly lower than those (*h*5 [−0.28 {0.40}mm], *h*5/*h*1[−0.03 {0.04}]) in the control group (*P* < .05) (Fig. 4). Above pulse parameters were closely related to arterial elasticity and peripheral resistance, the peripheral arterial resistance increased, and the vascular compliance decreased when above pulse parameters changed.

### 3.2. “Clinical symptoms - different periods” 2-mode network

**Figure 5.**
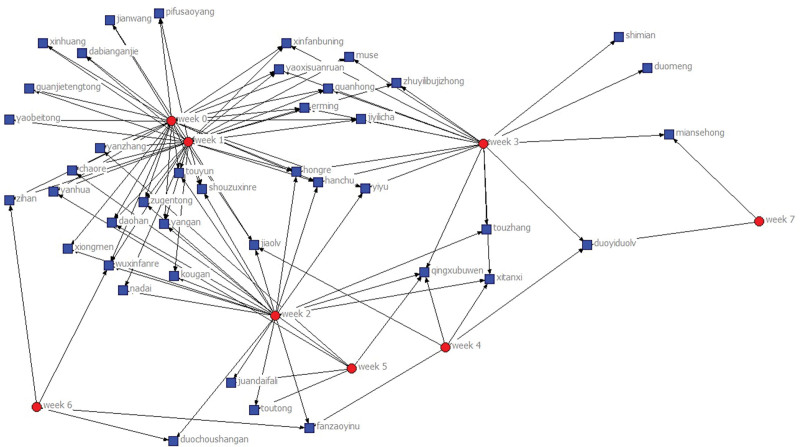
“Four diagnostic information - different periods” 2-mode network diagram.

We establish the 2-module network based on the gamma matrix (8 × 42). Since the subordinate network often uses 1 and 0 to indicate whether the relationship is established or not, it is necessary to binarize the data. In this study, the mean value of all values (1.52%) is taken as the segmentation value for binarization. If it is greater than 1.52%, it is taken as 1, otherwise it is 0. The changes of symptoms in different periods can be seen from the 2-mode network visualization map based on “different periods - symptoms” (Fig. 5), circular nodes represent different periods, square nodes represent symptoms. At week 0, there were 27 symptom information. The clinical symptoms of the patients were reduced continuously after treatment with RSRN, and there were 6 symptom information at week 5; the results suggested that the method of RSRN could effectively improve the clinical symptoms of patients and had obvious therapeutic effect.

### 3.3. Complexion index results before and after RSRN treatment

**Figure 6.**
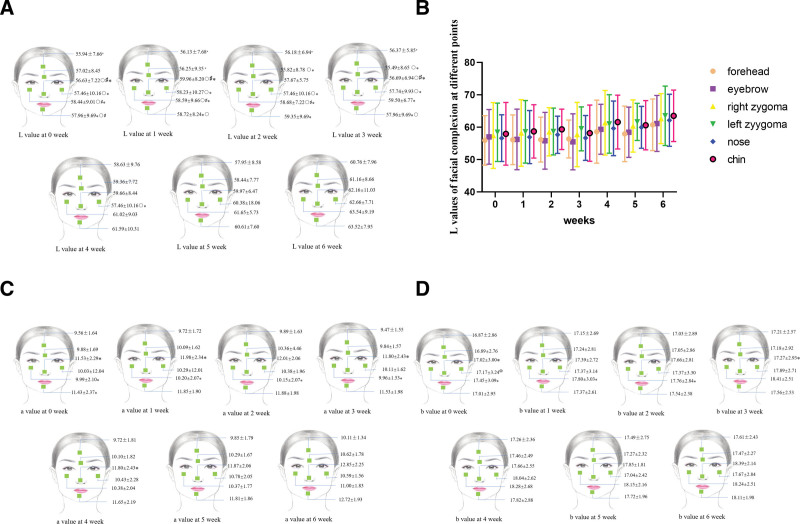
Complexion index results of the patients before and after RSRN treatment. (A) Complexion index *L* value of the forehead, eyebrows, left and right zygoma, nose, and chin at week 6 was significantly higher than that at weeks 0, 1, 2 ,3; (B) Changes of patients’ complexion index *L* value of the forehead, eyebrows, left and right zygoma, nose, and chin at different weeks based on statistical chart; (C) *a* value of the complexion index of zygomatic region, nose, and mandible at week 6 was significantly higher than that at weeks 0, 1; (D) *b* value of the complexion index of the left zygoma and nose at week 6 was significantly higher than that at week 0. ^○^*P* < 0.05, vs 4 weeks; ^#^*P* < 0.05, vs 5 weeks; ^⁎^*P* < 0.05, vs 6 weeks. The pictures were collected from zhidao. baidu).

The facial color index of patients had significant changes after the treatment of RSRN. The complexion index *L* of the forehead, eyebrows, left and right zygomatic, nose and mandible at week 6 was significantly higher than that at weeks 0, 1, 2, 3 (*P* < .05) (Fig. 6A, B); The *a* value of complexion index of zygomatic region, nose, and mandible at week 6 was significantly higher than that at weeks 0, 1 (*P* < .05) (Fig. 6C); the *b* value of complexion index of the left zygoma and nose at week 6 was significantly higher than that at week 0 (*P* < .05) (Fig. 6D).

### 3.4. Pulse condition and wave parameters before and after RSRN treatment

**Figure 7.**
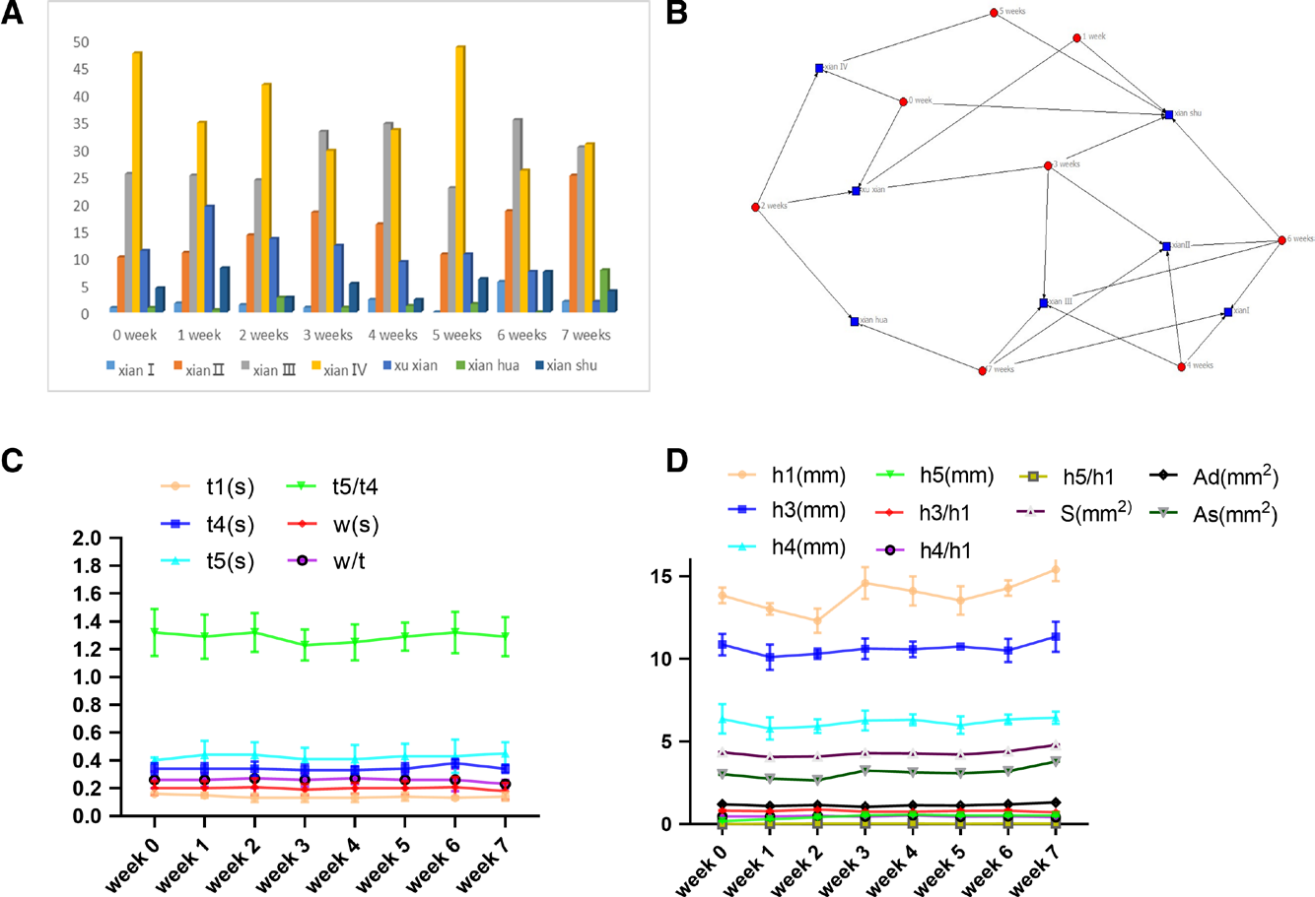
Pulse condition and wave parameters before and after RSRN treatment. (A) Changes of patients’ pulse condition at different weeks based on statistical bar chart; (B) Changes of patients’ pulse condition at different weeks based on 2-mode network visualization map; (C) Changes of patients’ pulse parameters *t*1, *t*4, *t*5, *t*5/*t*4, *w, w*/*t* at different weeks based on line chart; (D) Changes of patients’ pulse parameters *h*1, *h*3, *h*4, *h*5, *h*3/*h*1, *h*4/*h*1, *h*5/*h*1, *S, As, Ad* at different weeks based on line chart.

The pulse conditions were mainly expressed as string III (xian III) and string IV (xian IV) from week 0 to week 7. However, the pulse conditions including string III, string IV and xu xian were declined, and string II (xian II) and string slip (xian hua) were increased (Fig. 7A). It was mainly string IV pulse, xu xian pulse at week 0. After RSRN treatment, the pulse condition of patients was mainly string II, string slip at week 7 (Fig. 7B).

The *t*1 value of pulse parameter at week 1 was significantly higher than that at week 6; the pulse parameter *t*5 value at week3 was significantly lower than that at week7; The w and w/t value of pulse parameters at week 2 were significantly higher than those at week 7 (*P* < .05) (Table 1, Fig. 7C). The *h*1 value of pulse parameter at week 7 was significantly higher than that at weeks 1, 2. The *h*5 value of pulse parameter at week 7 was significantly higher than that at weeks 0, 1, 2, 3, 4, 5 (*P* < .05). The *h*3/*h*1 value of pulse parameter at week 7 was significantly lower than that at week 2. The pulse graph parameter *h*5/*h*1 at week 7 was significantly higher than that at weeks 0, 1, 2, 3, 4, 5. The *S* value of pulse parameter at week 7 was significantly higher than that at weeks 1, 2. The As value of pulse parameter at week 7 was significantly higher than that at weeks 0, 1, 2 (*P* < .05) (Table 2, Fig. 7D). The results suggested that the patient’s body function is improved, the healthy qi was enriched, and the elasticity of the great artery and the function of the aortic valve were also enhanced accordingly after treatment with RSRN.

**Table 1:** Pulse parameters of patients at different weeks (mean [SD]).

Weeks	*t*1 (s)	*t*4 (s)	*t*5 (s)	*t*5/*t*4 (s)	*W* (s)	*W*/*t*
0 week	0.15 (0.04)	0.34 (0.04)	0.44 (0.10)	1.32 (0.37)	0.20 (0.05)	0.26 (0.09)
1 week	0.15 (0.02)*	0.34 (0.04)	0.44 (0.10)	1.29 (0.36)	0.20 (0.05)	0.26 (0.05)
2 week	0.13 (0.03)	0.34 (0.05)	0.44 (0.09)	1.32 (0.34)	0.21 (0.07)†	0.27 (0.08)†
3 week	0.13 (0.03)	0.33 (0.04)*	0.41 (0.08)†	1.23 (0.31)	0.19 (0.04)	0.26 (0.05)
4 week	0.13 (0.03)	0.33 (0.03)	0.41 (0.10)	1.25 (0.33)	0.20 (0.04)	0.27 (0.04)
5 week	0.14 (0.03)	0.34 (0.04)	0.43 (0.09)	1.29 (0.30)	0.20 (0.05)	0.26 (0.06)
6 week	0.13 (0.03)	0.38 (0.03)	0.43 (0.12)	1.32 (0.45)	0.21 (0.07)	0.26 (0.08)
7 week	0.14 (0.03)	0.34 (0.03)	0.45 (0.08)	1.29 (0.24)	0.19 (0.06)	0.25 (0.03)

Note:

* *P* < 0.05, compared with 6 weeks;

† *P* < 0.05, compared with 7 weeks.

**Table 2:** Pulse parameters of patients at different weeks (mean [SD]).

Week	*h*1 (mm)	*h*3 (mm)	*h*4 (mm)	*h*5 (mm)	*h*3/*h*1	*h*4/*h*1	*h*5/*h*1	*S* (mm^2^)	*As* (mm^2^)	*Ad* (mm^2^)
0 week	13.85 (5.48)	10.87 (4.65)	6.37 (2.89)	0.52 (0.70)†	0.82 (0.31)	0.48 (0.23)	0.03 (0.05)†	4.35 (1.74)	3.13 (1.24)†	1.21 (0.66)
1 week	13.02 (5.35)†	10.11 (4.77)	5.80 (2.67)	0.42 (0.74)†	0.79 (0.30)	0.47 (0.22)	0.03 (0.08)†	4.06 (1.71)†	2.95 (1.23)†	1.10 (0.61)
2 week	12.31 (4.73)†	10.31 (4.31)	5.93 (2.42)	0.40 (0.66)†	0.88 (0.41)†	0.51 (0.29)	0.04 (0.06)†	4.10 (1.42)†	2.96 (1.04)†	1.15 (0.58)
3 week	14.59 (5.96)	10.62 (4.63)	6.27 (2.60)	0.54 (0.86)†	0.77 (0.41)	0.46 (0.20)	0.04 (0.06)†	4.30 (1.85)	3.24 (1.32)	1.06 (0.66)
4 week	14.11 (4.88)	10.58 (4.47)	6.32 (2.33)	0.56 (0.93)†	0.76 (0.22)	0.54 (0.82)	0.04 (0.06)†	4.29 (1.45)	3.14 (1.06)	1.14 (0.55)
5 week	13.54 (4.86)	10.76 (5.06)	6.00 (2.53)	0.53 (0.74)†	0.80 (0.29)	0.45 (0.14)	0.03 (0.05)†	4.20 (1.55)	3.08 (1.14)	1.12 (0.53)
6 week	14.29 (5.47)	10.52 (3.70)	6.34 (2.29)	0.35 (0.47)	0.82 (0.56)	0.48 (0.25)	0.02 (0.03)	4.40 (1.62)	3.20 (1.20)	1.21 (0.63)
7 week	15.41 (4.70)	11.35 (4.91)	6.44 (2.37)	0.19 (0.77)	0.73 (0.18)	0.42 (0.09)	0.01 (0.04)	4.80 (1.77)	3.50 (1.20)	1.31 (0.66)

Note:

*P < 0.05, compared with 6 weeks;

† *P* < 0.05, compared with 7 weeks.

### 3.5. Tongue condition and color parameters before and after RSRN treatment

**Figure 8.**
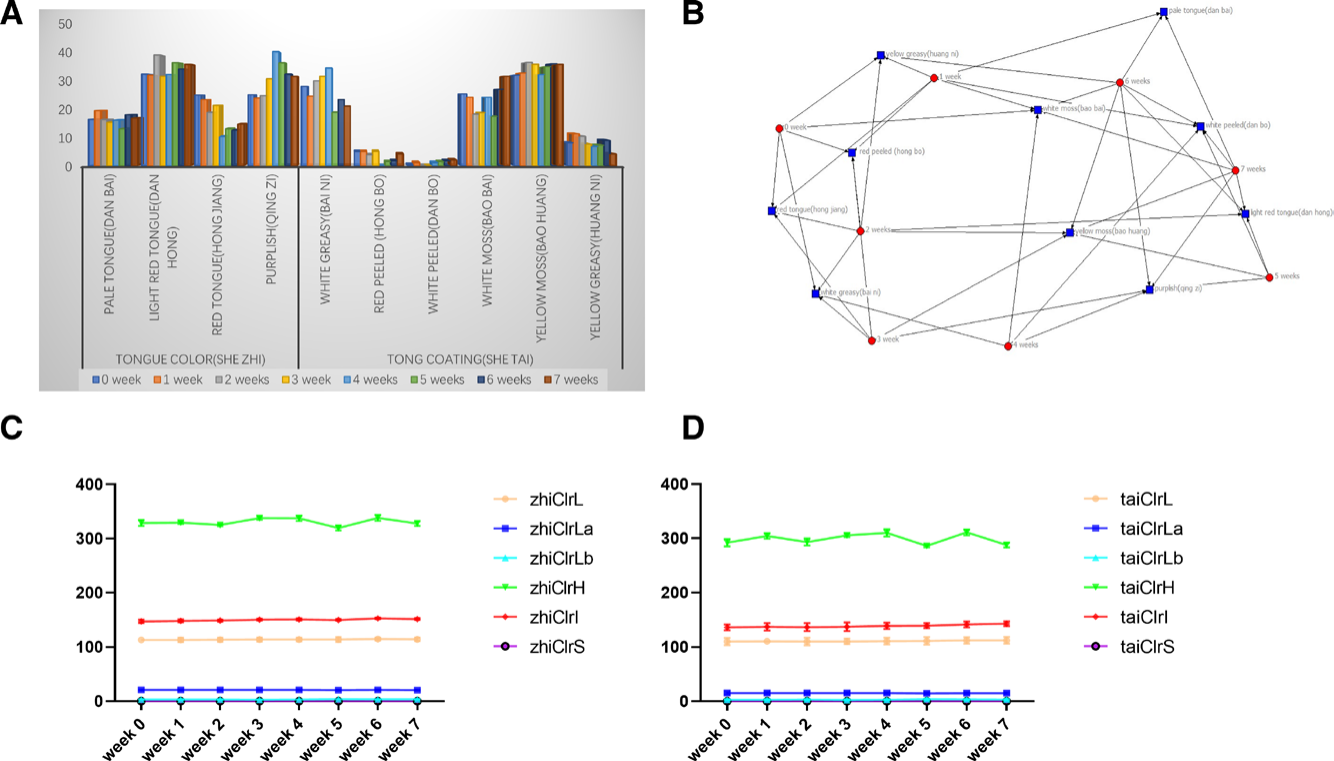
Tongue condition and color parameters before and after RSRN treatment. (A) Changes of patients’ tongue condition at different weeks based on statistical bar chart; (B) Changes of patients’ tongue condition at different weeks based on 2-mode network visualization map; (C) Changes of patients’ tongue color parameters Zhi *L, a, b, H, I, S* at different weeks based on line chart; (D) Changes of patients’ pulse parameters tongue color parameters Tai *L, a, b, H, I, S* at different weeks based on a line chart.

After the treatment of RSRN, the tongue image of the patients was purplish and reddish (she zhi); the tongue coating is greasy and peeling from week 0 to week 3; From week 4 to week 6, the tongue image of the patients was light red and light white (she zhi); the tongue coating is yellow and white (Fig. 8A).

Based on the 2-mode network display constructed by “tongue image - different periods” (Fig. 8B),we could see that the tongue image of patients at weeks 0, 1, 3 showed deep-red or purplish tongue (she zhi), and the tongue coating was mainly greasy and peeling; from week 4 to week 7, greasy fur and peeling fur were getting better, the tongue image was developing in a good way; the patient’s tongue showed thin white tongue, white greasy fur at week 4; at week 5, the patient’s tongue showed light red tongue, light yellow fur; the patient’s tongue showed light white tongue, thin white or yellow fur at weeks 6, 7.

The zhi L, tai *L* value of tongue color parameters at weeks 6, 7 were significantly higher than those at weeks 0, 1 (*P* < .05); and the zhi I, tai *I* value of tongue parameters at weeks 6, 7 were higher than those at weeks 0, 1 (Tables 3, 4; Fig. 8C, D).

**Table 3:** Tongue index of patients at different weeks (mean [SD]).

Week	ZhiClrL	zhiClrLa	zhiClrLb	zhiClrH	zhiClrI	zhiClrS	zhiCon	zhiENT	zhiMEAN
0 week	113.01(4.25)*,†	20.74(2.3)	3.24(2.14)	328.91(85.35)	147.33(13.03)*,†	0.16(0.02)	37.21(21.50)	1.03(0.11)	0.018(0.005)
1 week	113.23 (3.97)*,†	20.87 (2.27)	3.11 (2.39)	329.59 (82.54)	148.23 (12.52)*,†	0.17 (0.02)	37.59 (22.41)	1.03 (0.10)	0.018 (0.004)
2 week	113.41 (3.67)	20.85 (2.20)	3.32 (2.33)	325.09 (91.02)	148.73 (11.81)	0.16 (0.02)	36.41 (19.17)	1.02 (0.10)	0.017 (0.004)
3 week	113.89 (3.47)	20.89 (2.08)	2.91 (2.11)	337.96 (64.10)	150.45 (10.75)	0.16 (0.02)	39.15 (17.58)	1.03 (0.11)	0.018 (0.004)
4 week	113.99 (3.16)	20.87 (2.24)	3.17 (2.16)	337.48 (64.83)	150.81 (9.87)	0.16 (0.02)	37.47 (16.62)	1.04 (0.10)	0.018 (0.005)
5 week	113.84 (4.29)	20.43 (2.23)	3.80 (3.07)	319.51 (66.21)	149.94 (11.45)	0.16 (0.02)	34.82 (1.23)	1.02 (0.11)	0.017 (0.005)
6 week	114.73 (2.54)	20.83 (2.22)	3.43 (2.11)	338.18 (66.43)	152.96 (8.14)	0.16 (0.02)	36.61 (16.25)	1.03 (0.09)	0.019 (0.005)
7 week	114.31 (3.03)	20.61 (2.26)	3.46 (2.43)	327.86 (67.27)	151.59 (9.47)	0.16 (0.02)	35.95 (15.66)	1.03 (0.10)	0.018 (0.004)

Note:

* *P* < 0.05, compared with 6 weeks;

† *P* < 0.05, compared with 7 weeks.

**Table 4:** Tongue index of patients at different weeks (mean [SD]).

Week	taiClrL	taiClrLa	taiClrLb	taiClrH	taiClrI	taiClrS	taiCon	taiENT	taiMEAN
0 week	110.21 (6.77)*,†	15.08 (2.29)	2.97 (2.17)	292.13 (117.64)	136.30 (20.35)*,†	0.13 (0.02)	46.22 (23.31)	1.06 (0.12)	0.02 (0.006)
1 week	110.46 (6.44)*,†	15.20 (2.27)	2.99 (2.35)	304.34 (114.90)	137.11 (19.56)*,†	0.13 (0.02)	44.78 (27.56)	1.06 (0.14)	0.02 (0.005)
2 week	110.21 (6.94)	15.12 (2.16)	3.12 (2.41)	293.23 (116.50)	136.40 (21.40)	0.13 (0.02)	46.20 (23.19)	1.05 (0.15)	0.019 (0.005)
3 week	110.22 (8.12)	15.17 (2.29)	2.61 (2.21)	305.65 (113.15)	137.18 (21.88)	0.12 (0.02)	42.03 (21.43)	1.05 (0.16)	0.019 (0.005)
4 week	110.98 (6.10)	15.07 (2.07)	2.96 (2.08)	310.22 (106.73)	138.85 (18.54)	0.12 (0.03)	43.34 (20.56)	1.06 (0.12)	0.020 (0.005)
5 week	111.23 (6.77)	14.60 (1.95)	3.78 (3.25)	286.18 (112.21)	139.29 (20.57)	0.12 (0.02)	38.78 (20.79)	1.04 (0.15)	0.019 (0.005)
6 week	112.01 (5.71)	14.82 (1.92)	3.38 (2.27)	311.05 (109.13)	141.60 (17.31)	0.12 (0.02)	41.81 (18.74)	1.05 (0.15)	0.019 (0.005)
7 week	112.30 (6.20)	14.92 (2.42)	3.24 (2.59)	287.98 (111.32)	142.81 (19.22)	0.12 (0.02)	42.50 (21.52)	1.06 (0.12)	0.020 (0.005)

Note:

* *P* < 0.05, compared with 6 weeks;

† *P* < 0.05, compared with 7 weeks.

## 4. Discussion

Postmenopausal women have insufficient kidney essence, disharmony between Chong and Ren; when kidney yin is exhausted, the kidney water fails to restrict the heart fire and leads to a hyperactive heart fire; meanwhile, the heart fire burns essence into blood stasis, and congestion blocks pulse channels, which constitute the pathogenesis of postmenopausal women’s arteriosclerosis, therefore, the pathogenesis of postmenopausal arteriosclerosis can be summarized as “yin-deficiency of liver and kidney is the root of the disease, and excessive heart fire is the sign “.^[22]^ Based on the previous research,^[23,24]^ it was considered that the foundation of AS disease treatment lies in reducing the south (clearing the heart and removing blood stasis) and reinforcing the north (tonifying the kidney and essence) (RSRN). Pharmacological studies of RSRN involved anti-free radical effects,^[25]^ enhancing the function of hypothalamic pituitary gonadal axis^[26,27]^ and improving immune function.^[28]^

The 4 diagnostic methods of TCM (inspection, auscultation and olfaction, interrogation, and palpation) are important methods for clinical diagnosis and syndrome differentiation. It is the characteristic and advantage of TCM to evaluate human health through the four diagnostic information. In this study, the efficacy of the RSRN treatment of postmenopausal atherosclerotic diseases was evaluated by using the objective indicators of the TCM 4 diagnostic methods. Firstly, the pulse parameters between the disease group and the control group were compared and analyzed. The results showed that the peripheral resistance of arterial vessels in the disease group increased, the vascular compliance decreased, and the hardening of arterial wall changed. Secondly, the changes of patients’ symptoms, complexion, and tongue condition were observed before and after treatment with RSRN. The results of this study showed that RSRN could improve the clinical symptoms of patients. After treatment with RSRN, the patient’s complexion, pulse condition, and tongue condition were obviously improved.

It can be seen from the 2-model network of “clinical symptoms - different periods” that from 0 week to 7 weeks, the clinical symptoms of patients decreased significantly, it suggested that RSRN method can improve the clinical symptoms of postmenopausal AS patients. It was said that Qi comes from the viscera, and the color changes with the Qi in the classic book of TCM “Si Zhen jue wei.” The color of the human face is closely related to the internal organs, when the visceral function is abnormal, the human face is dull, dark, and spotted. After the treatment of RSRN, the *L, a*, and *b* values of facial color at different parts of patient’s face have significantly changed, and the overall chromaticity and brightness of the patient’s face have been improved, it was suggested that after the RSRN treatment, the skin color of the patient’s face was bright and moist, and the function of viscera had turned to a better direction.

Studies believed that the pulse of postmenopausal atherosclerotic patients was mainly string pulse, and the string pulse was mainly caused by the decrease of arterial elasticity, the increase of vascular wall tension, or the increase of peripheral resistance, and the decrease of arterial compliance.^[29]^ In this study, pulse parameters *t*1, *t*5, *w, w*/*t, h*3/*h*1, and *h*5/*h*1 related to peripheral vascular resistance changed to the trend of improvement after RSRN treatment. The result suggested that the elasticity of the great artery and the function of the aortic valve were enhanced accordingly after treatment with RSRN. The patient’s tongue image also changed greatly after RSRN treatment, and it was mainly characterized by purplish tongue or dark-red tongue, peeling or greasy fur at week 0; after the treatment of RSRN, the patient’s tongue image was characterized by light red tongue, thin yellow, or thin white fur. There were also significant changes in *L* and *I* index of tongue image, which showed that the brightness of tongue texture and tongue coating increased, it suggested that the dark purple of tongue texture was better than before.

The clinical treatment of diseases in TCM is mainly based on symptoms, emphasizing the combination of four diagnosis and treatment based on syndrome differentiation. The observation of TCM diagnosis and their dynamic changes is an important prerequisite for guiding the TCM clinical treatment. This study started with the combination of disease and syndrome - accurate diagnosis and treatment, and attempted to observe the 4 diagnostic indicators and their dynamic changes of postmenopausal atherosclerotic disease patients before and after the treatment of RSRN through the analysis of symptoms, digital tongue image, digital pulse data, and digital facial color data. The mechanism of its influence on the occurrence and development of postmenopausal atherosclerotic diseases may be related to its regulation of blood lipid metabolism, correction of arterial tension and compliance; The changes of the four diagnostic information can provide new indicators for the clinical efficacy evaluation of postmenopausal atherosclerotic diseases, and provide new ideas and methods for the establishment of a clinical efficacy evaluation system with Chinese characteristics.

In conclusion, the method of RSRN can improve the complexion, tongue, pulse, and clinical symptoms of postmenopausal patients with AS. Objective indicators of the 4 diagnostic methods of TCM can be used to evaluate the clinical treatment effect, to guide the clinical treatment of TCM.

## Acknowledgments

The authors thank all the women and research team members who participated in this study.

## Author contributions

**Conceptualization:** Hongjin Wu, Zhifeng Zhang.

**Data curation:** Hongjin Wu, Weiwei Dai, Zhifeng Zhang.

**Funding acquisition:** Shenguang Li.

**Investigation:** Shenguang Li.

**Methodology:** Jiatuo Xu.

**Project administration:** Jiatuo Xu.

**Software:** Liping Tu.

**Supervision:** Liping Tu.

**Writing—original draft:** Hongjin Wu.

**Writing—review & editing:** Zhifeng Zhang.
